# Pfarao: a web application for protein family analysis customized for cytoskeletal and motor proteins (CyMoBase)

**DOI:** 10.1186/1471-2164-7-300

**Published:** 2006-11-29

**Authors:** Florian Odronitz, Martin Kollmar

**Affiliations:** 1 Department of NMR-based Structural Biology, Max-Planck-Institute for Biophysical Chemistry, Am Fassberg 11, 37077 Goettingen, Germany

## Abstract

**Background:**

Annotation of protein sequences of eukaryotic organisms is crucial for the understanding of their function in the cell. Manual annotation is still by far the most accurate way to correctly predict genes. The classification of protein sequences, their phylogenetic relation and the assignment of function involves information from various sources. This often leads to a collection of heterogeneous data, which is hard to track. Cytoskeletal and motor proteins consist of large and diverse superfamilies comprising up to several dozen members per organism. Up to date there is no integrated tool available to assist in the manual large-scale comparative genomic analysis of protein families.

**Description:**

Pfarao (Protein Family Application for Retrieval, Analysis and Organisation) is a database driven online working environment for the analysis of manually annotated protein sequences and their relationship. Currently, the system can store and interrelate a wide range of information about protein sequences, species, phylogenetic relations and sequencing projects as well as links to literature and domain predictions. Sequences can be imported from multiple sequence alignments that are generated during the annotation process. A web interface allows to conveniently browse the database and to compile tabular and graphical summaries of its content.

**Conclusion:**

We implemented a protein sequence-centric web application to store, organize, interrelate, and present heterogeneous data that is generated in manual genome annotation and comparative genomics. The application has been developed for the analysis of cytoskeletal and motor proteins (CyMoBase) but can easily be adapted for any protein.

## Background

The success of the genome sequencing projects have culminated in release 149 of GenBank [1] that announced two milestones: the total sequence data passed the 100 gigabases mark, and, for the first time, the number of bases derived from whole genome shotgun sequencing projects exceeded the number of bases in the traditional divisions of GenBank. However, the process of genome annotation still lags considerably behind that of genome data generation. Although many tools have been developed for the *ab initio *annotation of whole genomes, especially the annotation of data from higher eukaryotes yields low success rates [2]. The success rates can considerably be increased by similarity searches of EST data or of annotated data from other genomes. But also these data have their drawbacks: ESTs are fragmentary and might suffer from several artefacts including contamination with genomic DNA; similarities to proteins in other species might suffer from evolutionary divergence or the orthologue-paralogue problem [3]; and the presence of alternative splicing considerably complicates the interpretation of alignments between genomic DNA, cDNAs and ESTs. More seriously, however, similarity data is never complete. But it is the annotation that connects the sequence to the biology of the organism [4].

Manual annotation is still by far the most accurate and successful way to achieve correct predictions of genes. This process is best done using the possibilities of comparative genomics and multiple sequence alignments. Because a majority of the proteins are not characterized and their functions are largely unknown, the initial process involves categorizing these predicted proteins into subsets of proteins or protein families based on homology, presence of various functional domains and motifs, as well as similarity to well characterized proteins from other species.

Thus, when working with collections of protein-sequences from different species and sources, one quickly accumulates large amounts of heterogeneous data: Protein and DNA sequences, their identifiers in different databases, references to literature, information about species including taxonomy, and links to online resources like sequencing projects. Since data that can be retrieved from public databases is often incomplete or incorrect it is very desirable to be able to combine manually edited with automatically generated content. In addition, there is often misleading and contradicting data, especially concerning the nomenclature and classification of proteins, that needs to be tracked and commented.

Cytoskeletal and motor proteins have extensively been studied in the past. They are involved in diverse processes like cell division [5], cellular transport [6], neuronal transport processes [7], or muscle contraction [8], to name a few. Especially motor proteins consist of large superfamilies. E.g. vertebrates contain up to 60 myosins and about the same number of kinesins that are spread over more that a dozen distinct classes. Since genome sequence data is rapidly accumulating it is very important to have a reference database for the nomenclature and phylogenetic relation of the proteins that allows the most accurate assignment of biological function possible.

Pfarao is a database driven web application that was written to assist researchers investigating structure, function and phylogeny of proteins. It has been developed for the analysis of cytoskeletal and motor proteins (CyMoBase), but can be adapted to any type of protein. It stores, organizes, interrelates, presents, and analyzes data of various sources. Additionally, it triggers external prediction programs, so that manually entered and automatically generated data is always synchronized.

## Construction

### Technologies

The system is running on UNIX (OS X and Linux) systems. The database management system is PostgreSQL [9]. As web application framework we chose Ruby on Rails [10] since it has the advantage of rapid and agile development while keeping the code well organized. Part of this framework is an implementation of Active Record [11] which is an O/RM (Object-relational Mapping) system that makes database integration into an object oriented program considerably easier. This also allows to use the interactive ruby shell (irb) with database rows wrapped in objects for interaction with the database. This way of accessing the data often proves superior to the SQL shell. Additionally, Ruby on Rails offers XML-RPC so data can be accessed by other programs.

We implemented a service-oriented mechanism that starts specific scripts, when records in the database are added or updated. In this case, a PostgreSQL trigger starts a PL/Ruby script [12], which opens a network connection to a delegation server program written in Distributed Ruby [13] on the same machine and calls one of its functions, giving a database ID as an argument if appropriate. The server can in turn start scripts to act upon the entered or updated data and returns after completion so that the database transaction is completed. The server's state can be set from within the database or from external programs to disable certain functions during batch processing in order to avoid flooding.

The automation scripts for parsing BLAST [14] and HMMER [15] output are written in Ruby [16] making use of the BioRuby library [17]. Sequences are scanned for domains using the Pfam_fs release 19.0 database [18] containing 8183 hidden markov models.

The web pages are generated as XML (XHTML with SVG [19] data islands). We used SVG [19] for charts because of the high display quality and the possibility of reuse in print. The site makes extensive use of Ajax (Asynchronous JavaScript and XML) in order to present the user with a feature rich interface while minimizing the amount of transferred data. All technologies used are freely available and open source.

### Database

The unique requirements of the system demand a custom database schema. The schema is sequence-centric with an additional emphasis on species since these two aspects are the most important in mutual annotation and, therefore, need to be represented in high detail (see additional file 1: Database schema). Grouped around these central tables are tables for literature and sequencing projects as well as taxonomy and predicted domains.

The sequence table stores the protein sequence and the corresponding sequence as derived from the multiple sequence alignment of the protein (see Import/Export). By relating a position in the alignment to the positions in a set of protein sequences it is possible to retrieve homologous stretches from different sequences. In addition there are fields for sequence classification and nomenclature, comments, legacy names, information about the completeness of the sequence, its potential to be a pseudo-gene, and links to records in NCBI's nucleotide and protein databases [20]. The comment field is one of the most important fields intended to contain information about differences of the database sequences to published sequences that may have resulted from wrong exon predictions or sequencing errors. Records in the sequence table are related to tables for proteins, species, and publications.

Several versions can be assigned to each sequence so changes and corrections can be tracked as more information becomes available. Furthermore, there are links to tables containing automatically generated protein domain predictions (see Automated processes).

Species are defined by a set of names. There are fields for the scientific name of a species, the species abbreviation as used to identify database sequences, and common names. As some species are known by different scientific names, fields containing alternatively used names are also included. To account for the different usage of the scientific names, all possible names are listed and linked to the corresponding reference record wherever species are listed or used for selection via the interface. A comment field may contain general information about the corresponding species, the specific strain used, or common and divergent features compared to closely related organisms. The taxonomy field is converted automatically into a hierarchical representation of the taxa. (see Automated processes)

Proteins are stored with their name and abbreviation as used in the database. Furthermore, classes of a certain protein can be grouped and categorized according to aspects like cellular function or localization. The project table includes information about the sequencing centres including type of data and completeness. Publications can be related either to a sequence to provide additional links to biological information or to a sequencing project.

Data entry is done using the iiwi system (Odronitz F., Lampetsdoerfer T., Dietrich D., unpublished results [21]) allowing for remote editing and access control.

### Automated processes

**Figure 1:**
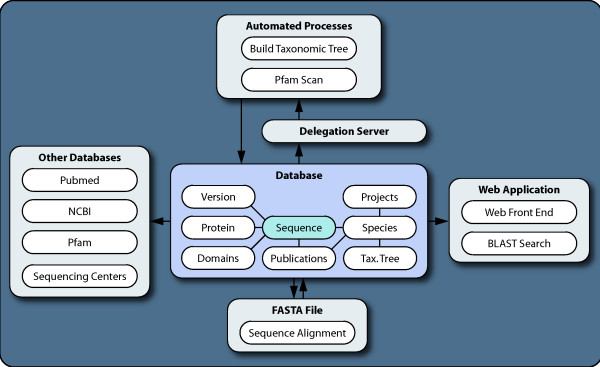
Diagram of main tables and linked resources. The Database (blue) with the central sequence table (cyan), important associated tables (white) and connected systems are shown. Lines connecting tables depict table relations. Arrows depict flow of information. FASTA files containing sequence alignments are imported and exported using Ruby [16] scripts. The import function uses the BioRuby [17] library. Other databases are referenced via their IDs, which are used to generate hyperlinks to records on their web sites. Automated processes are started by a delegation server, which receives instructions from the database on insert or update of records. The automated processes write information into the database using Active Record. The frontend is generated using the Ruby on Rails [10] web application framework.

**Figure 2:**
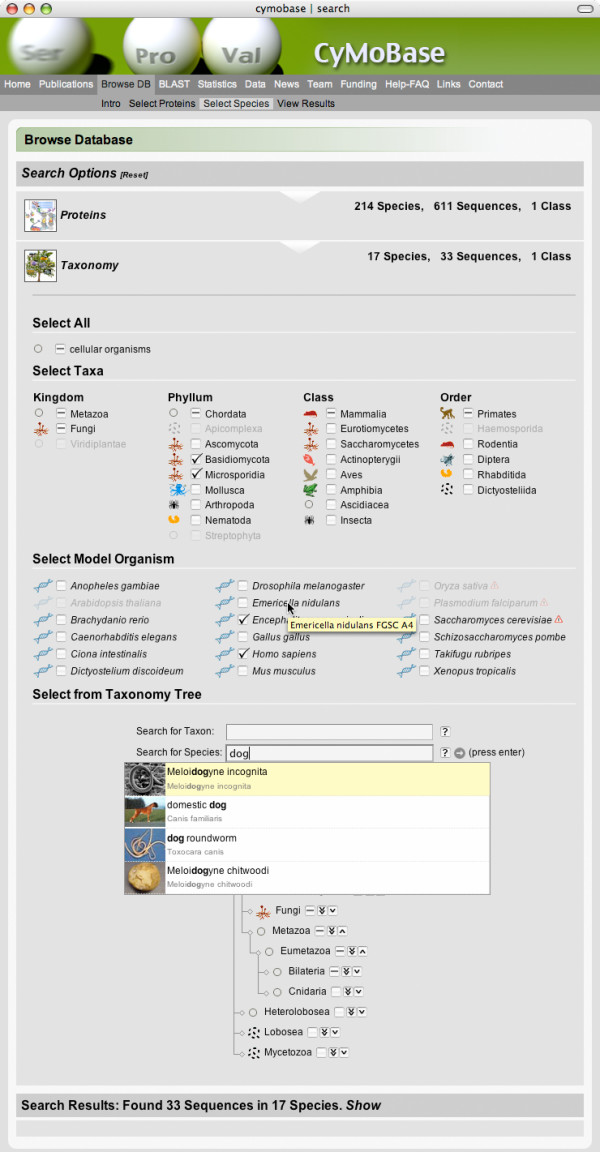
Screenshot of the species selection interface. The user can select all species or a subset of species. Taxa and species for which no sequences for the selected proteins/protein classes exist are greyed out (taxa selection, model organisms) or are invisible (tree). Each node of the tree can be expanded and collapsed. The auto-completion fields open and highlight the tree down to the taxon/species typed. Common names like "dog" are also supported. All sections of the page respond to changes. Example: Nothing is selected. User selects kingdom Fungi. This selects all phyla, classes, orders, species and model organisms that belong to Fungi. Also the portion of the tree below Fungi is selected. User deselects Ascomycota. All elements react accordingly. User selects Homo sapiens. User clicks 'Show' in the result section and is presented with a list of sequences from Homo sapiens and all Fungi, excluding Ascomycota (see Fig. 3).

The database can trigger external programs upon insertion or update of certain fields in the database tables by contacting the delegation server program, which can in turn write computed data to the database (Fig. 1). When a protein sequence is inserted or changed a hmmpfam [22] process is started scanning this sequence for putative domains with Pfam [18] profiles. The obtained domain identifier and the start and end positions together with the E-value are stored in a database table. Upon insertion of a new species record, the content of the taxonomy field is automatically converted into a tree-representation of interrelated taxon records. Each record contains the name of the taxon, and a reference to the parent taxon. Then the species record is connected to the common taxonomic tree. This tree representation of the taxonomy allows for convenient searching, browsing and selection of sub-trees (Fig. 2).

### Import/export functions

Files containing protein sequences in FASTA-format can be imported into the database to update existing or insert new records in the sequence table (Fig. 1). A naming convention at all levels ensures the correct assignment of sequences in a FASTA file to sequence records in the database. The sequence identifiers are a concatenation of species name abbreviation, protein name abbreviation, protein class and protein variant. In contrast to the usage of numerical database IDs, the naming convention thus immediately provides the user with information about the phylogenetic relation and possible functions of the protein. Sequences and sequence alignments can be exported from the database using filters to include only certain proteins, protein classes, or sequences from species in certain taxa. The resulting FASTA file also follows the naming convention and therefore can be re-imported after editing. Thus it is possible to retrieve a multiple sequence alignment from the database, edit it manually and write it back to the database. During import, sequences with identifiers that do not match any record in the database, induce the creation of a new database record according to the information included in the identifier.

## Utility and discussion

The requirements for Pfarao can be summarized as follows: The key component of the database is the protein sequence that is obtained by manual annotation of genome and EST data with the help of a multiple sequence alignment. The sequence needs to be connected to data that allows the useful interpretation of the results concerning its biological function, and it needs to be linked with primary databases like GenBank or PubMed. To be useful for the specific protein community, whose members are expected to work in all biological and medical sub disciplines, the information of the database has to be presented in the most comprehensible way.

### Web interface

Great attention has been paid to a versatile yet easy to use web interface. We think that accessibility and high quality representation is key to a productive usage of the system.

Data can be entered and edited using a series of forms and lists. Relations are represented as pull-down menus.

Pfarao encompasses a live web front end that is generated from the content of the database at each request and thus always reflects the current data, eliminating the need for manual updates. To browse the content of the database, the user selects a set of proteins and protein classes, and is then guided to refine the selection by choosing a set of specific taxa or species. Taxa and species can either be selected from tables containing specific subsets, or from a tree representation of the taxa and species that is generated to match the protein and protein class selection. Taxa and species can be browsed and selected by expanding/collapsing and including/excluding subsections of the tree, or by using shortcuts or auto-completion fields (Fig. 2). We consider the selection of specific species and taxa a key feature for comparative analyses of protein inventories and diversity (Fig. 2).

**Figure 3:**
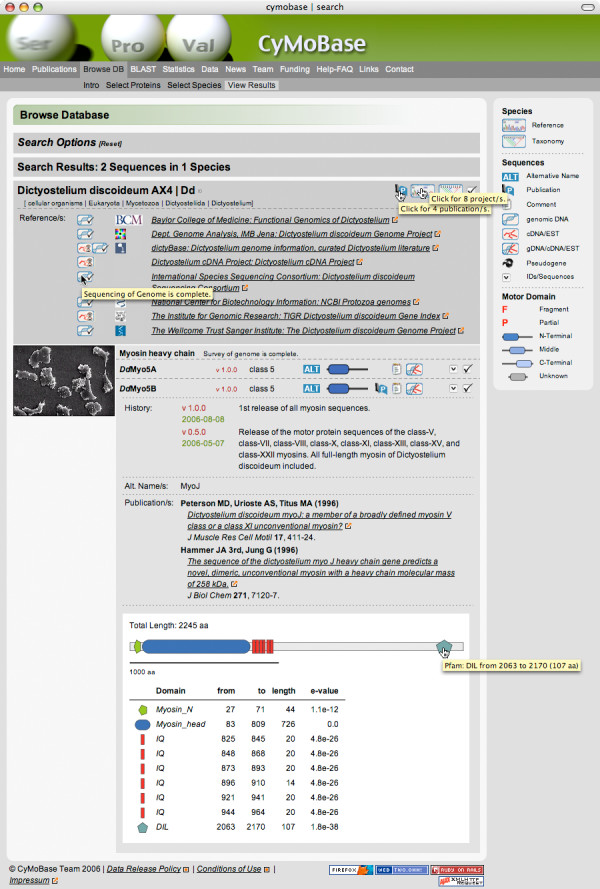
Screenshot of the protein sequence view. The list is grouped by species. Sequences are ordered by the protein name. Different types of information are available for each species (publications, references to sequencing projects, taxonomy and name information) and each protein sequence (version history, alternative names, domain composition, publications, comments, source, amino acid sequence, links to other databases). All the details can be shown (and hidden) selectively. This way, even long lists can be viewed without cluttering the page. The data is retrieved on demand from the server via Ajax and does not have to be downloaded to the user's computer if not needed. Cursor labels provide the user with a short summary of the information behind the icons. A click shows the complete information. Additional cursors added to the figure to show cursor labels.

Upon confirmation of the selection of protein and species, the system compiles a list of all sequences matching the specified criteria and presents it as a list grouped by species in taxonomic order. Additional data about the species like alternative names, links to sequencing centres and publications, as well as detailed information about the sequences including publications, comments, domain organization, and the sequence data, can selectively be shown or hidden (Fig. 3).

The system provides an integrated BLASTP [14] search and is able to link the sequences in the BLAST database with the records in the SQL database via an ID. Thus the user can, apart from the sequence, immediately access all related information. The organization of the database lends itself to different types of statistical analysis. For each protein, a set of tables and graphs can be generated. These analyses provide important information for the comparison of the protein inventory of specific taxa and species, as well as important insights into the selected protein superfamily. The protein inventory table gives an overview about the class distribution and the number of class members of all or a number of selected species (ordered by taxonomy). Color-coding of the cells helps to quickly identify characteristic patterns of specific taxa. Charts show the ratio of protein classes and the distribution of the molecular weight for a chosen set of classes. All charts are generated on the fly in resolution-independent SVG-code, so they can also be used for print.

### Future developments

Pfarao provides a solid platform for additional features and significant future developments of the system are underway. The front end will be extended to allow the graphical representation and fast browsing of large alignments of selected sequences that will be of great value for mutational studies. The interface is also intended to support the generation of phylogenetic trees for a user-defined set of sequences. These extensions will increase the transparency of the manual annotation process, as the user will be able to look at the two basic sources of information about protein sequence relations. It is also planned to incorporate the corresponding DNA data and to track the various alternative splice forms of the proteins.

### Case study

Pfarao has initially been developed for cytoskeletal and motor proteins but can easily been adapted to any protein. The database for cytoskeletal and motor proteins is called CyMoBase [23]. Our current in house database contains 3265 Sequences (3095759 amino acids) from 666 species, 494 publications, and 385 references to 165 sequencing projects but is being extended on a daily basis. A portion of the data has been released in the publicly available CyMoBase.

## Conclusion

Here, we introduce a web application for the analysis of proteins from manual annotation and their relationship. The major motivation for this work was to provide an integrated environment that organizes and relates all relevant information and presents it using a high quality interface. Pfarao is a tool that allows the researcher to constantly monitor the state of the work without having to manually aggregate data from a range of sources. It has been developed for the analysis of cytoskeletal and motor proteins (CyMoBase) but can easily be customized for any type of protein.

## Availability and requirements

CyMoBase can be accessed at .

Due to the technologies used, it requires Firefox version 1.5 or greater with cookies and JavaScript enabled. Other browsers do not have the required feature set or do not comply with the standards of the W3C [19]. The database schema, the web application, the server program and all scripts can be obtained upon request and used under a Creative Commons License. Use of Pfarao by non-academics requires permission.

## Abbreviations

ID Identification Number

SVG Scalable Vector Graphics

XHTML Extensible HyperText Markup Language

XML Extensible Markup Language

XML-RPC Extensible Markup Language – Remote Procedure Call

## Authors' contributions

MK specified the requirements from a users perspective, defined the rules for data handling and participated in the design of the interface. He collected all the data and evaluated every function of the system. FO carried out the implementation of the system, designed the database scheme and did the technical design and the programming. Both authors wrote and approved the final manuscript.

## Supplementary Material

Additional File 1 The file contains the detailed database schema. Click here for file
