# Supplementary material for: Pfarao: a web application for protein family analysis customized for cytoskeletal and motor proteins (CyMoBase)
Source: BMC Genomics. 2006 Nov 29;7:300. doi: 10.1186/1471-2164-7-300 (PMC1684263; doi:10.1186/1471-2164-7-300)
Supplement: Additional File 1 — The file contains the detailed database schema. [file 1471-2164-7-300-S1.pdf]

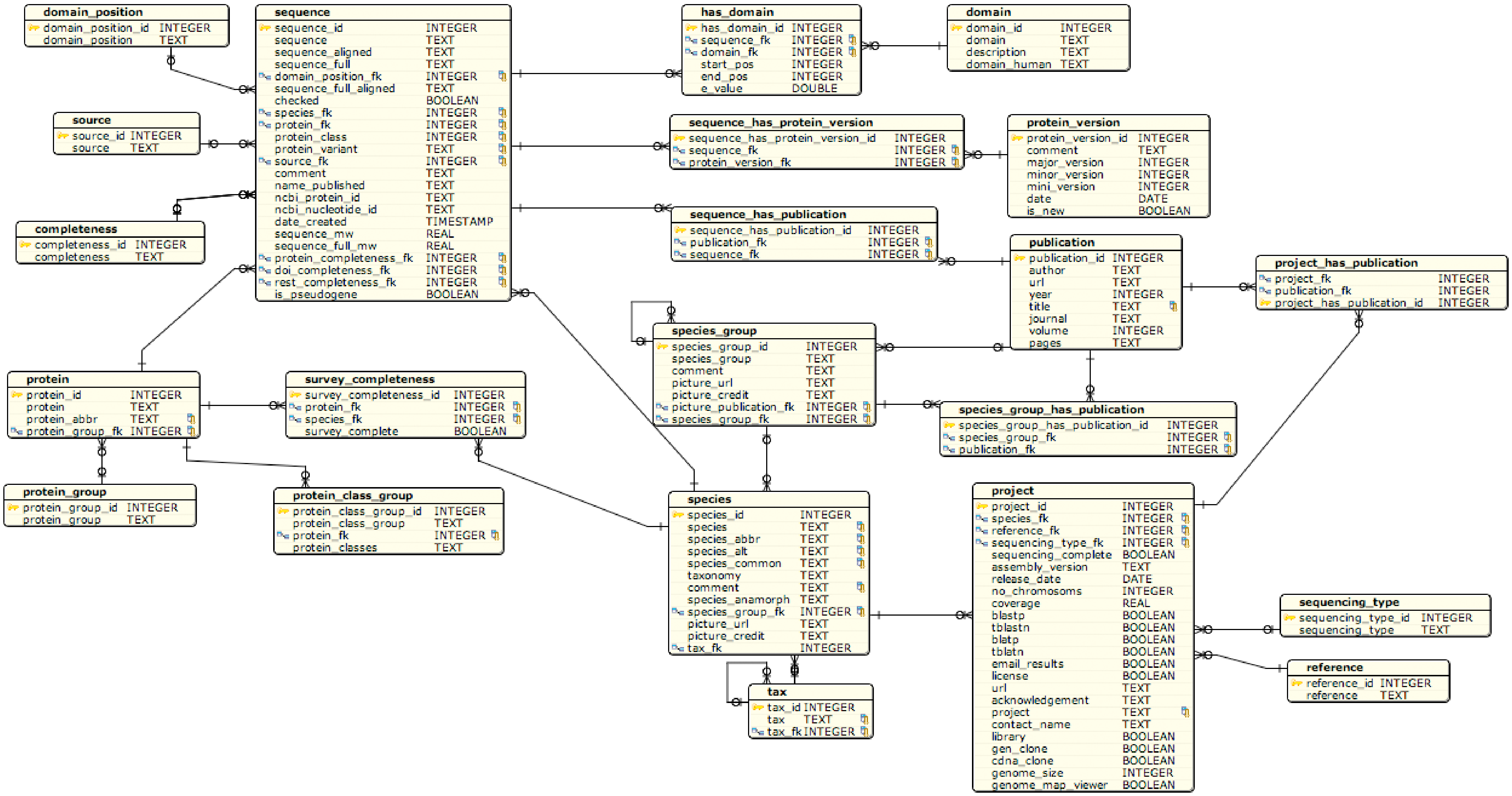

Supplementary Figure 1.

Database schema. The schema shows the database tables and their relations. For each table the columns are listed with their name and datatype. Yellow keys in front of the names signify columns with unique identifiers. Blue window-symbols mark foreign key columns that contain values of id-columns of other tables. Symbols at the right side of the column names designate indices for better performance. Lines are relations between tables. Two unary (recursive) relationships are defined: One linking taxa to their parent taxon and one linking species groups to their parent group.
